# Exosomes Derived from BM-MSCs Mitigate the Development of Chronic Kidney Damage Post-Menopause via Interfering with Fibrosis and Apoptosis

**DOI:** 10.3390/biom12050663

**Published:** 2022-05-02

**Authors:** Wardah A. Alasmari, Ahmed Abdelfattah-Hassan, Hanaa M. El-Ghazali, Samar A. Abdo, Doaa Ibrahim, Naser A. ElSawy, Eman S. El-Shetry, Ayman A. Saleh, Mohammed A. S. Abourehab, Hala Mahfouz

**Affiliations:** 1 Department of Anatomy, Faculty of Medicine, Umm Al-Qura University, Makkah 24230, Saudi Arabia; 2 Department of Anatomy and Embryology, Faculty of Veterinary Medicine, Zagazig University, Zagazig 44519, Egypt; dr_h1980@hotmail.com; 3 Biomedical Sciences Program, University of Science and Technology, Zewail City of Science and Technology, Giza 12578, Egypt; 4 Department of Biochemistry, Faculty of Veterinary Medicine, Zagazig University, Zagazig 44519, Egypt; samer_ahmed289@yahoo.com; 5 Department of Nutrition and Clinical Nutrition, Faculty of Veterinary Medicine, Zagazig University, Zagazig 44511, Egypt; doibrahim@vet.zu.edu.eg; 6 Department of Human Anatomy and Embryology, Faculty of Medicine, Zagazig University, Zagazig 44511, Egypt; naser_elsawy@ymail.com (N.A.E.); emanelshetry@zu.edu.eg (E.S.E.-S.); 7 Department of Animal Wealth Development, Genetics & Genetic Engineering, Faculty of Veterinary Medicine, Zagazig University, Zagazig 44519, Egypt; lateefsaleh@yahoo.com; 8 Department of Pharmaceutics, College of Pharmacy, Umm Al-Qura University, Makkah 21955, Saudi Arabia; maabourehab@uqu.edu.sa; 9 Department of Medical Biochemistry and Molecular Biology, Faculty of Medicine, Kafrelsheikh University, Kafrelsheikh 33516, Egypt; hala_ali2015@med.kfs.edu.eg

**Keywords:** exosome therapy, renoprotective, nephroprotective, chronic renal injury, caspase 3, fibrosis

## Abstract

The rate of chronic kidney disease (CKD) is increasing globally, and it is caused by continuous damage to kidney tissue. With time the renal damage becomes irreversible, leading to CKD development. In females, post-menopause lack of estrogen supply has been described as a risk factor for CKD development, and studies targeting post-menopause CKD are scarce. In the present study, we used exosomes isolated from bone marrow mesenchymal stem/stromal cells (BM-MSCs) to test their therapeutic potential against the development of CKD. At first, the menopause model was achieved by surgical bilateral ovariectomy in female albino rats. After that, 100 µg of exosomes was given to ovariectomized rats, and the study continued for 2 months. Changes in urine volume, urine protein content, kidney function biochemical parameters (creatinine and BUN), kidney antioxidant parameters (SOD, GPx and CAT), histological changes, immunohistochemical levels of caspase 3, and the gene expression of NGAL (related to kidney damage), TGFβ1 and αSMA (related to fibrosis and EMT), and caspase 3 (related to apoptosis) were studied. After the ovariectomy, the occurrence of CKD was confirmed in the rats by the drastic reduction of serum estrogen and progesterone levels, reduced urine output, increased urinary protein excretion, elevated serum creatinine and BUN, reduced GPx SOD, and CAT in kidney tissue, degenerative and fibrotic lesions in the histopathological examination, higher immunohistochemical expression of caspase 3 and increased expression of all studied genes. After exosomes administration, the entire chronic inflammatory picture in the kidney was corrected, and a near-normal kidney structure and function were attained. This study shows for the first time that BM-MSCs exosomes are potent for reducing apoptosis and fibrosis levels and, thus, can reduce the chronic damage of the kidneys in females that are in their menopause period. Therefore, MSCs-derived exosomes should be considered a valuable therapy for preserving postmenopausal kidney structure and function and, subsequently, could improve the quality of females’ life during menopause.

## 1. Introduction

Chronic kidney disease (CKD) is characterized by the gradual loss of kidney functions and an increased risk for death. The global prevalence of CKD is estimated to be 11–13%, and most affected patients are in stage 3 [1]. The available data also revealed that females are more prone to CKD compared to males [1]. Seven percent of postmenopausal females with normal kidney function will develop CKD within 10 years (https://www.mayoclinicproceedings.org/article/S0025-6196(20)30374-8/fulltext, last accessed on 5 April 2022). CKD will gradually progress toward end-stage renal failure, which is fatal and requires dialysis or kidney transplantation and causes life-threatening and financial problems for patients and health systems. Various factors contribute to the occurrence of CKD (such as chemicals, medications, genetics, immunity, infections, environment and diet). The association between menopause and CKD was given less attention compared to other conditions, and limited data can be found related to CKD and menopause [2]. In fact, the role of menopause in the development of CKD is complex, and associated with various predisposing factors, such as aging, cardiovascular diseases, abnormal mineral metabolism and increased oxidative stress in the body, most of these conditions are related to the lack of estrogen during post-menopause (reviewed in detail in [2]). The role of renal fibrosis and the involvement of TGF-β in the development of CKD has been extensively studied [3,4], and the inhibition of TGF-β can lead to favorable outcomes in terms of the prevention of fibrosis in CKD [5]. Another important aspect of CKD development is the apoptosis of renal glomerular and tubular cells [6,7], with caspases, and essentially caspase 3, playing a pivotal role in the progression of CKD [8]. Therefore, therapeutic approaches that target both TGF-β and caspase 3 should be beneficial in preventing the development of CKD in post-menopause females.

It has been investigated that decreased estrogen levels during menopause lead to significant effects on the female body. In addition, it predisposes them to other significant diseases, such as bone mineralization and cardiovascular diseases, which are under heavy investigation lately. However, no data exists on the targeting of post-menopause CKD. As a result of the lack of estrogen, it is widely accepted that hormone replacement therapy can be effective in menopausal females; however, hormone therapy results, in general, were described to be controversial [9]. In addition, in vivo studies showed that supplying estrogen can lead to reduced renal damage and oxidative stress in ovariectomized rats [10]. However, several reports showed that hormonal replacements are not recommended for prolonged use, as long treatment was proven to cause adverse effects, including kidney damage [11]. Consequently, post-menopause CKD is still lacking appropriate therapeutic approaches to reduce the rate of apoptosis and fibrosis, which occur in the kidneys, leading to their chronic damage, and there are currently no known treatment regimens. 

Recently, studies have elucidated that mesenchymal stem cells (MSCs) possess therapeutic capabilities for many diseases, mainly through their paracrine mechanisms [12,13,14]. These paracrine actions are mediated by the release of soluble factors and encapsulated factors (inside microvesicles and exosomes). The latter is of special importance since exosomes released from MSCs are responsible for the horizontal transfer of important mRNA, microRNA and proteins and are now being recognized as an integral component of intercellular communication [15,16,17]. Exosomes are ideal therapeutic agents because their complex content of proteins and genetic materials has the potential to treat complex diseases, such as CKD. In addition, exosome-based therapy circumvents some of the concerns and limitations of using viable replicating cells, including mesenchymal stem cells [18]. One of the main contributors to kidney regeneration is stem cells that reside in the kidney itself (reviewed in [19]). However, isolated stem cells from animals suffering from CKD showed decreased regenerative potential and premature senescence [20]. The latter study shows that during CKD, the kidney regenerative capacity was not functioning properly and contributes to advancing chronic kidney damage. Very recently, adipose MSCs and their secreted vesicles were found to be beneficial in protecting against ischemia-reperfusion acute kidney failure [21]. However, there is a global concern about using viable replicating cells, and much clinical investigation is still needed; therefore, stem cells’ secreted exosomes come as a promising cure for diseases with complex etiology, such as CKD in postmenopausal females. In this regard, exosomes used for treating acute kidney injury have drawn recent attention [22,23,24]. In addition, few studies reported the use of exosomes for treating chronic kidney injury (reviewed in [25]). 

In the literature, there is a lack of studies that focus on the use of exosomes derived from BM-MSCs for reducing the progression of CKD during menopause, and there is limited information on the use of exosomes or their renoprotective effects during post-menopause. Therefore, the current study intended to investigate the therapeutic efficacy of exosomes released from MSCs and their use as a novel therapeutic approach to prevent/reduce apoptosis and fibrosis associated with chronic kidney damage in postmenopausal females.

## 2. Materials and Methods

### 2.1. Study Animals and Experimental Design

For this study, a group of healthy female albino rats (n = 28) of similar age and weight (approx. 7 months old and their weight averaged 300 g approx.). The rats were obtained from the animal house of the Faculty of Medicine, Zagazig University, and were housed in a temperature/humidity-controlled (24 ± 2 °C and 50 ± 10% relative humidity) and light-controlled room (12 h light/dark cycle) with free access to a standard rat chow diet and filtered tap water. The rats were permitted to acclimatize for 2 weeks before starting the experiment. All experimental procedures followed the guidelines of the Institutional Animal Care and Use Committee of Zagazig University (protocol number: ZU-IACUC/2/F/164/2020), and the study was conducted in strict accordance with the recommendations in the Guide for the Care and Use of Laboratory Animals of the National Institutes of Health (NRC, Washington D.C., USA). The control group contained 7 healthy female rats, while the remaining rats were randomly assigned to one of the following groups: Sham group, included rats that underwent the same operation as the ovariectomy group but with no ovariectomy (Sham, n = 7), post-menopausal chronic kidney damage group (CKD, n = 7), and, finally, CKD rats that received exosomes derived from BM-MSCs (CKD + Exosomes, n = 7). The administration of MSCs-derived exosomes was performed within 24 h after ovariectomy or sham surgical procedures, and the study continued for 2 months to assure the occurrence of CKD in operated rats.

### 2.2. Isolation and Characterization of Bone Marrow MSCs

Rat bone marrow was collected from the long bones of 4–6 week-old, apparently healthy rats, as previously reported by others and us [26,27]. The process included, briefly, collecting long bones and the flushing of their bone marrow using a syringe containing 10,000 IU heparin to prevent coagulation. The collected bone marrow was pooled and was cultured on tissue culture plates. With frequent medium changes, the non-adherent cells were washed off; the medium was changed after 3 h from initial plating and then every 8 h during the succeeding 72 h. The remaining cells were only adherent cells that are considered to be mesenchymal stem cells (MSCs). The characterization of cultured MSCs was performed to confirm that these cells were MSCs; this was done by flow cytometric analysis of FITC-labeled anti-CD34 (Cat. No: 555821, BD Pharmingen, San Diego, CA, USA), anti-CD45 (ab33916, abcam, Boston, MA, USA), anti-CD90 (ab226, abcam, USA) and anti-CD105 (ab184667, abcam, USA) antibodies, as we previously reported [28]. MSCs used for exosomes isolation were from passages 4 to 7. 

### 2.3. Isolation and Characterization of Exosomes from BM-MSCs 

Exosome isolation, characterization (by TEM and western blotting) and storage were performed as previously reported by us and others [27,29]. In brief, starting from passage three, the MSCs culture medium was removed when the cells reached 80% confluency, and the serum-free medium was replenished and left for 24 h; it was collected and now called conditioned medium (CM). The collected CM was filtered through a 0.22 μm filter (to remove debris and dead cells) and was centrifuged at 10,000 g for 30 min at 4 °C and the supernatant was collected. The supernatant was then subjected to ultracentrifugation at 100,000 g for 60 min at 4 °C to pellet the exosomes. The pellet was washed twice with ice-cold PBS, and ultracentrifugation was performed for each wash to re-pellet the isolated exosomes. To quantify the amount of isolated exosomes, the protein content was measured by standard Bradford assay, and then the isolated exosomes were adjusted to 100 µg protein in 200 µL aliquots and stored at −80 °C. The characterization of isolated exosomes was performed using the detection of exosomes’ markers, CD63 (sc-5275, Santa Cruz Biotechnology, USA) and CD81 (sc-166029, Santa Cruz Biotechnology, Dallas, Texas, USA), by western blotting and by transmission electron microscopic (TEM) detection of their shape and size. 

### 2.4. Post-Menopause Chronic Kidney Disease (CKD) Model

The establishment of the post-menopause CKD model followed previously published protocols [30,31]. Briefly, strict aseptic surgical procedures were performed; after that, the assigned rats were anesthetized using intraperitoneal injection of a mixture of Ketamine (80 mg/kg) and Xylazine (12.5 mg/kg). A 20 mm dorsal skin incision was cut in the midline to allow access for two 10mm flank muscle incisions to excise both ovaries from both sides. After the removal of the ovaries, the incisions were sutured and the rats were observed for complete recovery. Administration of analgesia (midazolam 1 mg/kg s.c.) and antibiotics (gentamicin 5 mg/kg i.m.) was performed for 5 days post-surgery. The sham-operated rats underwent the same surgical procedures except for the ovaries, which were not removed. The success of the operation was confirmed by the absence of monthly cycles and the abrupt decrease in serum levels of estrogen and progesterone. Other signs were reduced uterine mass observed during sample collection at the end of the study, as reported previously [32]. The rats were humanely euthanized by an overdose of inhalation general anesthesia at the end of the study.

### 2.5. Urine Analyses

Urine was collected in metabolic cages for a total period of 24 h at the end of the study (after 2 months of CKD induction). The urine analysis included the estimated glomerular filtration rate, calculated as previously reported [11], and the evaluation of urine protein content by standard Bradford assay kit (Pierce™ Coomassie (Bradford) Protein Assay Kit, ThermoFisher Scientific Inc., Waltham, MA, USA), following the manufacturer’s instructions.

### 2.6. Administration of Exosomes Derived from Bone Marrow MSCs

Exosomes purified from step 2.3 were administered intravenously through the tail vein in the CKD + Exosomes group; the rats received one-time 100 µg protein-equivalent of MSCs-derived exosomes in 200 µL. The injections were performed after the rats were completely recovered following CKD induction [24]. 

### 2.7. Serum Biochemical Assays and Kidney Tissue Antioxidants Detection

Samples from the rat’s blood were collected for serum separation. Serum samples were used to measure the levels of creatinine (Creatinine ELISA Kit, Catalog #: E4370, BioVision, Inc., USA), BUN (BUN ELISA Kit, Catalog #: MBS2611085, MyBioSource, USA), estrogen (Estrogen ELISA Kit, Catalog #: K4266, BioVision, Inc., USA) and progesterone (Progesterone ELISA Kit, Catalog #: K7416, BioVision, Inc., USA), this was performed by commercial kits and according to the kit’s recommendations. Moreover, kidney samples were collected immediately after sacrifice, washed twice in warm PBS, and then subjected to homogenization, as we previously reported [33]. The antioxidant markers evaluated in kidney tissue were the levels of superoxide dismutase (SOD), glutathione peroxidase (GPx) and catalase (CAT); their measurement was conducted using commercial kits and following the kit’s recommendations (Bio-diagnostics Co., Cairo, Egypt and BioVision, Inc., Milpitas, CA, USA).

### 2.8. Histopathologic Examination

After sacrificing the study rats, immediately, a kidney was collected and longitudinally excised and immersed in a fixative (10% neutral buffered formalin) solution. After 24–48 h, the fixed samples of kidney tissue were paraffin-embedded and histologically prepared by conventional histological technique, as previously described [33]. After that, tissue sections (4 µm) were stained using the standard H&E technique. The extent of renal damage was blindly evaluated by an independent pathologist.

### 2.9. Immunohistochemistry of Caspase 3

For the evaluation of caspase 3 expression in kidney tissue, paraffin-embedded kidney tissue was cut into 4 µm sections, and the sections were exposed to the standard heat-antigen retrieval procedure. Then, blocking serum was added (to block non-specific binding), followed by the addition of H_2_O_2_ (to block endogenous peroxidases). The anti-caspase 3 primary antibody was added (1:250, rabbit caspase 3 antibody, ab13,847, abcam, USA), and the slides were incubated overnight at 4 °C. The secondary antibody was added to the slides (1:500, HRP-labeled goat anti-rabbit antibody, ab6721, abcam, USA) and incubated for 30 min at room temperature. Finally, the DAB substrate was used for the visualization of positive reactions (Mouse and Rabbit Specific HRP/DAB Detection kit, ab64264, abcam, USA). The slides were examined under light microscopy, and staining intensity was determined by ImageJ software (ImageJ v 1.53, National Institutes of Health, Bethesda, MD, USA), as previously reported [28].

### 2.10. Real Time qPCR

Kidney tissue samples were immediately collected after sacrifice and were kept in Qiazol Lysis Reagent (Cat. No.: 79306, Qiagen, Cairo, Egypt) and stored at −20 °C. The RNA extraction from kidney tissue samples was performed using an RNA extraction kit (RNeasy Mini Kit, Cat. No.: 74106, Qiagen, Cairo, Egypt), following the manufacturer’s recommendations. The extracted RNA was checked for concentration and purity using nanodrop at 260 and 280 nm wavelengths (Quawell Q5000, Quawell Technology, Inc., San Jose, CA, USA). After that, reverse transcription into cDNA was performed using a RevertAid First Strand cDNA Synthesis Kit (Thermo Scientific, Cat. No. K1621, Cairo, Egypt). The expression of study genes (the used primers’ sequences are listed in Table 1) was performed using StepOnePlus™ Real-Time PCR system (Applied Biosystems, Waltham, MA, USA) by using QuantiTect SYBR^®^ Green PCR Kit (Qiagen, Cat. No. 204141, Cairo, Egypt). The resulting gene expression data were normalized against GABDH (as a housekeeping gene), and the amount of amplified products was relatively quantified using the ^−2ΔΔ^Ct method, as previously described.

### 2.11. Statistical Analyses

The obtained data from this study were analyzed using one-way ANOVA, followed by Tukey’s HSD between-group analysis using PASW statistical package (SPSS v18, SPSS Inc., Chicago, IL, USA). The gene expression data were analyzed by one-way ANOVA and visualized using GraphPad Prism 5 (GraphPad Software Inc., La Jolla, CA, USA). Statistical significance was considered when the *p*-value ≤ 0.05, and the data were presented as means ± SD.

## 3. Results

### 3.1. BM-MSCs Isolation and Collection of Their Exosomes 

**Figure 1:**
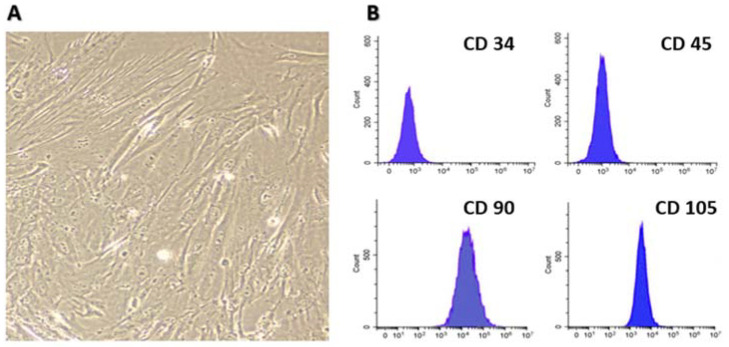
Characterization of isolated BM-MSCs. (**A**) Isolated BM-MSCs in culture at passage 3 with the typical fibroblast-like shape. (**B**) Flow cytometric evaluation of BM-MSCs markers seen as a positive expression of CD90 and CD105, and negative expression of endothelial CD34 and hematopoietic CD45 markers.

**Figure 2:**
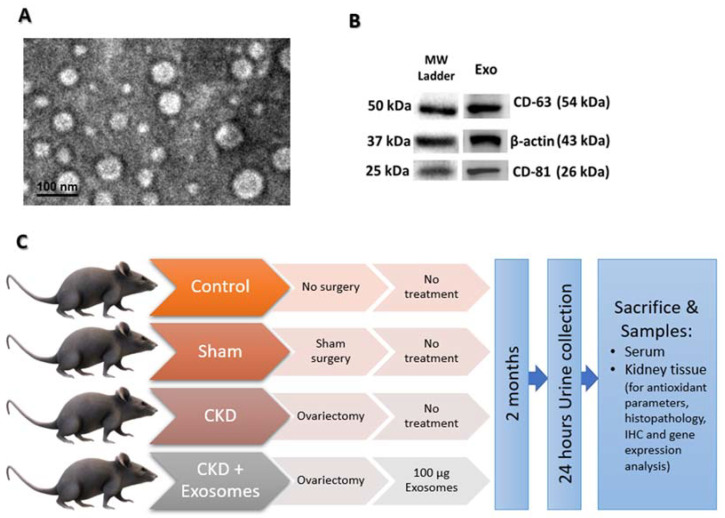
Characterization of exosomes obtained from BM-MSCs. (**A**) TEM examination showing the presence of exosomes and their diameter is less than 100 nm. (**B**) Western blot analysis of exosomes’ markers CD63 and CD81. (**C**) Scheme outlining the experimental setup of the study.

After the isolation of BM-MSCs the cells were characterized by their typical plastic adherence, fibroblast-like morphology (Figure 1A) and flow cytometric detection of specific MSCs marker (CD90 and CD105) and negative expression of endothelial and hematopoietic markers (CD34 and CD45, respectively), as shown in Figure 1B. Following the isolation of exosomes by ultracentrifugation, their examination by TEM revealed the appearance of nano-vesicles with an average diameter of 70 nm corresponding to exosomes (Figure 2A). In addition to TEM, the isolated exosomes were subjected to western blot analysis, and there was a positive expression of specific exosome markers (CD63 and CD81, Figure 2B). 

### 3.2. Changes in Serum Estrogen and Progesterone Levels in Study Rats

**Figure 3:**
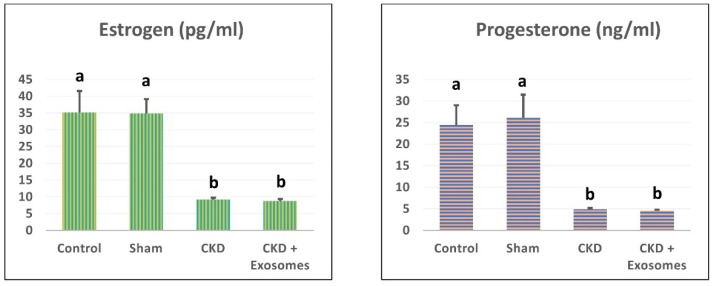
Changes in serum estrogen and progesterone levels in study rats. CKD + Exosomes group received an intravenous injection of 100 µg protein-equivalent of exosomes in their tail vein. Different letters denote statistical significance (*p* < 0.05, using Tukey’s HSD post-hoc test).

After the successful ovariectomy procedure and throughout the study, all ovariectomized rats showed a significant decrease in estrogen and progesterone levels (*p* < 0.05, Figure 3). In addition, there was no difference between the ovariectomy group or the group treated with exosomes. 

### 3.3. Changes in Serum/Tissue Biochemical Parameters 

**Figure 4:**
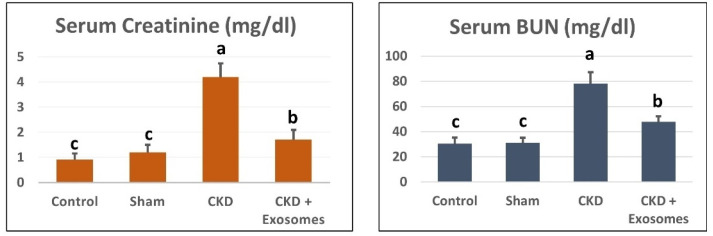
Serum biochemical parameters (creatinine and blood urea nitrogen) following treatment with MSCs-derived exosomes in study groups. The CKD + Exosomes group received an intravenous injection of 100 µg protein-equivalent of exosomes in their tail vein. Columns with different letters denote significant differences between groups (*p* < 0.05, using Tukey’s HSD post-hoc test).

**Figure 5:**
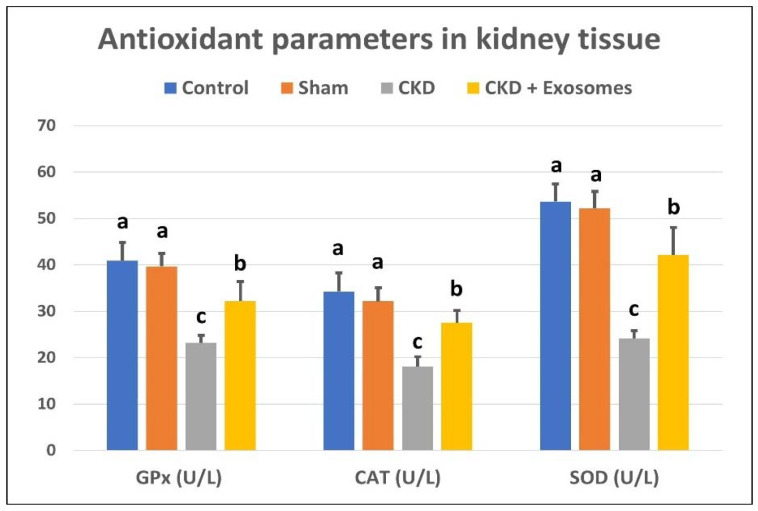
Antioxidant parameters in kidney tissue, glutathione peroxidase (GPx), catalase (CAT) and superoxide dismutase (SOD) in the different study groups. The CKD + Exosomes group received an intravenous injection of 100 µg protein-equivalent of exosomes in their tail vein. Columns with different letters denote significant differences between groups (*p* < 0.05, using Tukey’s HSD post-hoc test).

The serum levels of BUN and creatinine in ovariectomized rats were significantly increased (*p* < 0.01) compared to control and sham groups (Figure 4). In addition, the kidney-tissue levels of antioxidant parameters, SOD, GPx and CAT were significantly decreased (*p* < 0.01, Figure 5) in ovariectomized albino rats compared to the control or sham groups. Following the administration of exosomes isolated from BM-MSCs, serum levels of BUN and creatinine were significantly decreased (*p* < 0.01) compared to the ovariectomized group, but their levels were still higher than the control and sham groups. Moreover, kidney levels of SOD, GPx and CAT were partially restored (*p* < 0.01) to comparable levels to those in the control and sham groups.

### 3.4. Changes in GFR and Urinary Protein Excretion 

**Figure 6:**
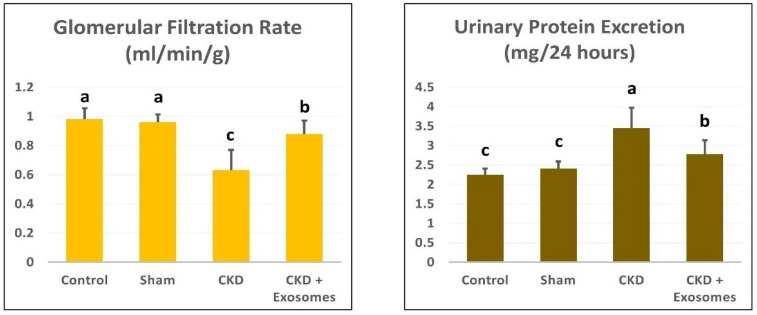
Changes in glomerular filtration rate (GFR) and urinary protein excretion in the different study groups. The CKD + Exosomes group received an intravenous injection of 100 µg protein-equivalent of exosomes in Their tail vein. Columns with different letters denote significant differences between groups (*p* < 0.05, using Tukey’s HSD post-hoc test).

Compared to the non-ovariectomized groups (control or sham), the ovariectomized group showed a significant decrease in the glomerular filtration rate and a significant increase in urinary proteins excretion (*p* < 0.01, Figure 6). Following exosome administration, the treated rats had restored their glomerular filtration rate and urinary protein excretion levels to near-normal levels.

### 3.5. Histological Changes in Kidney Tissue 

**Figure 7:**
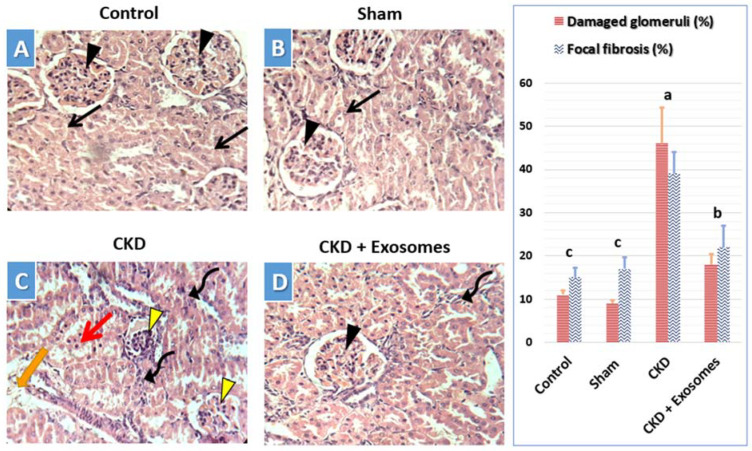
Representative micrographs for the histological evaluation of the lesions in the kidneys of different studied groups. (**A**) Control group, (**B**) Sham group, (**C**) CKD group, and (**D**) CKD + Exosomes group; The CKD + Exosomes group received an intravenous injection of 100 µg protein-equivalent of exosomes in their tail vein. Black arrow head = normal glomeruli, Yellow arrow head = shrunken glomeruli with degenerated glomerular cells, wide intraglomerular space and edema in some glomeruli, Black arrow = normal renal tubules, Red arrow = degenerated renal tubular epithelial cells, Orange arrow = fibrosis, and Curved arrow = mononuclear cells infiltration. The right bar graph shows the percentage of damaged glomeruli (shrunken with/without edema) and the percentage of focal fibrosis in the different study groups, columns with different letters denote significant differences between groups (*p* < 0.05, using Tukey’s HSD post-hoc test).

In the control (Figure 7A) and sham-operated (Figure 7B) groups, the observed histological structure of the kidney was apparently normal, with normal renal tubules and normal renal corpuscles. In the chronic kidney damage group (Figure 7C), the observed kidney’s histological structure indicated signs of chronic damage, including degenerated and shrunken renal corpuscles, which could show edema, in addition to dispersed necrotic renal tubules in between normal tubules. Moreover, perivascular mononuclear cells’ infiltration with fibrosis in some areas was also noticed in kidneys with chronic damage (Figure 7C). Following exosome administration, the study rats showed substantially less chronic damage signs and a more conserved kidney histological structure (Figure 7D). This includes apparently normal renal corpuscles with less frequent degenerative changes in both the corpuscles and tubules surrounding it. This was accompanied by a drastic reduction in mononuclear cells infiltration and fibrosis in the treated kidneys.

### 3.6. Detection of Caspase 3 Expression in the Kidney by Immunohistochemistry 

**Figure 8:**
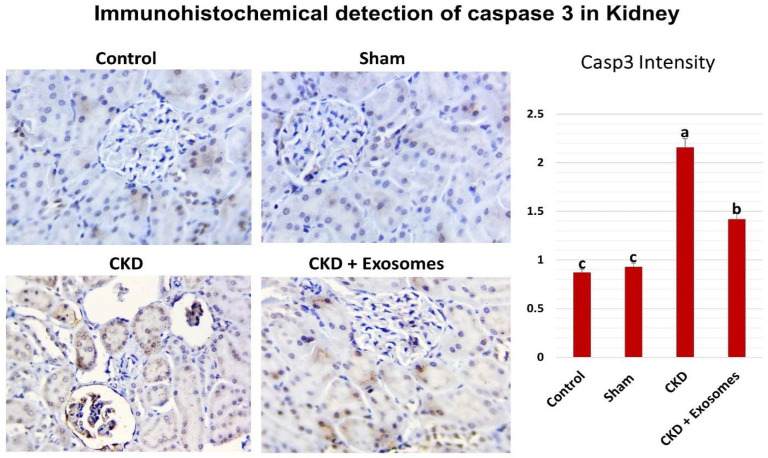
Representative micrographs showing the expression of caspase 3 in kidney tissue by immunohistochemistry in the different studied groups. The CKD + Exosomes group received an intravenous injection of 100 µg protein-equivalent of exosomes in their tail vein. The right bar graph shows the intensity of caspase 3 staining in the different groups, columns with different letters denote significant differences between groups (*p* < 0.05, using Tukey’s HSD post-hoc test).

The immunohistochemical detection of caspase 3 (shown in Figure 8) revealed no or very few cells that are positive for caspase 3 in both control and sham-operated groups with no significant difference between them. In the rats experiencing chronic kidney damage, there was a more diffuse and stronger expression of caspase 3 in kidney tissue in the glomeruli and renal tubules. Whereas caspase 3 levels were drastically reduced in the group receiving exosomes derived from MSCs, which was apparent in low caspase 3 expression in the glomeruli and reduced expression in the renal tubular cells.

### 3.7. Gene Expression Results by qPCR

**Figure 9:**
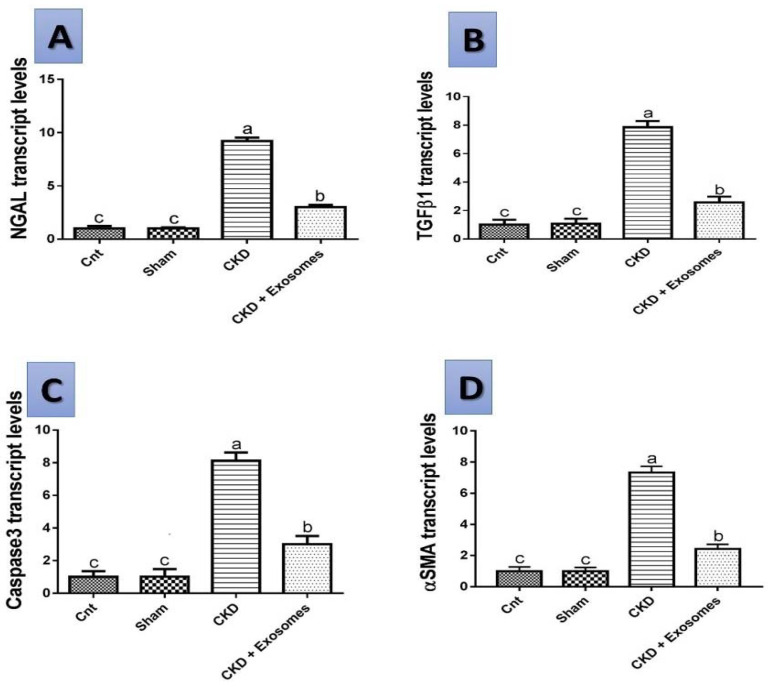
Changes in the mRNA expression of (**A**) neutrophil gelatinase-associated lipocalin (NGAL), (**B**) transforming growth factor beta 1 (TGFβ1), (**C**) caspase 3 and (**D**) smooth muscle alpha-actin (αSMA) genes in the different studied groups. The CKD + Exosomes group received an intravenous injection of 100 µg protein-equivalent of exosomes in their tail vein. Columns with different letters denote significant differences between groups (*p* < 0.05, using Tukey’s HSD post-hoc test).

The level of expression of the studied genes NGAL, TGFβ1, αSMA and caspase 3 in the different studied groups are shown in Figure 9. In the rats experiencing chronic kidney damage, there was an upregulation of the expression of all studied genes (*p* < 0.01) compared to either the control or sham groups. However, following exosomes administration, there was a marked downregulation of the genes’ expression to reach a level slightly higher than in the control or sham groups and significantly lower than the chronic kidney damage group (*p* < 0.05).

## 4. Discussion

The results obtained in this study show for the first time that MSCs-derived exosomes reduced both the apoptosis of renal cells and the fibrotic changes inside the kidney, thus slowing the progression of CKD in post-menopause. Reduced apoptosis was achieved via reducing caspase 3 mRNA and protein expression levels in kidney tissue. The diminished fibrotic changes were achieved by reducing the expression levels of TGFβ1 and αSMA together with less evident fibrotic changes in the histological examination of kidney tissue. Therefore, MSCs exosomes contribute to slowing the rate of chronic damage to the kidney during menopause in females. This was confirmed by the reduced expression of the NGAL gene, reduced serum creatinine and BUN as known markers for kidney damage. In addition, the kidney’s histological picture was significantly improved. Previous studies showed that exosomes could be used for the treatment of acute kidney injury [37]; however, less data are present on the use of exosomes for the treatment of chronic kidney injury [25]. Furthermore, no studies were reported on testing the therapeutic effects of BM-MSCs exosomes on CKD that are associated with post-menopause states in females.

The successful achievement of a post-menopause-like condition in the study rats following bilateral ovariectomy was confirmed by various indicators. The earliest signs were the observed hormonal changes (reduced estrogen and progesterone levels) and the absence of estrus cycles, while at slaughter, the visible degenerated uterine tissue are all good indicators, as previously reported [38,39,40,41]. After two months following menopause induction, CKD was evident, and its indicators were the marked increase in serum creatine and BUN, the increase in urinary protein excretion and the reduced urine output [42,43]. The chronic damage was further confirmed by the prominent histological changes in kidney histopathological examination that were visible as chronic mononuclear cells infiltration, shrunken glomeruli, degenerated renal tubular cells, focal areas of fibrosis and edema [44,45]. This was accompanied by reduced antioxidant reserve (GPx, SOD and CAT) in kidney tissue and altered gene expression of NGAL, TGFβ1, αSMA and caspase 3 genes that are related to CKD [35,46,47,48].

Neutrophil gelatinase-associated lipocalin (NGAL) is among the earliest markers that were found important to indicate renal damage [49]. In addition, serum NGAL levels were found to be positively correlated with the severity of kidney damage and are considered to be promising next-generation biomarkers for kidney diseases (reviewed in detail in [50]). In CKD in particular, NGAL was correlated with levels of serum creatinine, and glomerular filtration rate, and all three indicators reflected the extent of renal damage [51]. In the present study, the expression of the NGAL gene was increased in the rats of the CKD group, agreeing with previous studies. After the treatment with exosomes, the expression levels of NGAL were greatly reduced and approached the normal levels in non-CKD rats. These results clearly indicate a reduction in renal damage, which was confirmed by the biochemical and histological results of the current study. In addition, NGAL was considered to have a protective role against apoptosis by preventing the overexpression of caspase 3 in the kidney [52], as the suppression of NGAL expression by siRNA led to increased expression of caspase 3 and increased apoptosis of kidney cells. 

Caspase 3 is one member of the apoptosis-associated proteases that degrade intracellular components and lead to cell death. It is one of the final proteases to be activated before cell death and the formation of apoptotic bodies [53]. In addition, the inhibition of caspase 3 activation led to a reduction in both the rate of apoptosis and kidney damage and protected against acute renal failure [54]. In the present study, in the CKD group, the gene expression of caspase 3 was upregulated, as was the immunostaining of caspase 3, which was localized to both the tubules as well as the glomerular cells. Our results are in accordance with previous studies that showed that caspase 3 levels are associated with apoptosis, fibrosis and chronic renal scarring (i.e., fibrosis) and were detected in the tubular cells and glomeruli [8]. Following exosome administration, in the CKD + exosomes group, the expression levels of both caspase 3 mRNA and protein in kidney tissue were greatly reduced and approached the normal levels observed in the control and sham groups. The association between apoptosis, fibrosis and the progression of CKD was previously demonstrated where apoptosis of renal cells increased together with the rate of fibrosis in kidney tissue [7]. 

The fibrosis in kidney tissue, as well as in other tissues, is greatly mediated by the TGF-β pathway, and the inhibition of TGF-β is a major target for therapeutic approaches against chronic kidney disease associated with fibrosis [3,5] and other fibrotic diseases in general [55]. In the kidney, the inhibition of TGF-β signaling is also associated with decreased epithelial mesenchymal transition (EMT) and the accumulation of myofibroblasts in the kidney, thus reducing renal fibrosis and the progression of chronic kidney injury [56]. EMT is also implicated in the pathogenesis of CKD and is characterized by the expression of both TGF-β and αSMA [57], and the prevention of EMT is important for protecting renal tissue and reducing kidney damage. In our study, the expression of TGFβ1 was increased in the CKD group and was associated with the increased expression of αSMA (which is an EMT and myofibroblast marker) in kidney tissue and also associated with the prominent pathological lesions observed in the kidney histological examination, which included degeneration and fibrotic reactions. However, in the exosome-treated group, the expression levels of TGFβ1 and αSMA were significantly reduced and approached the control and sham rates; this indicates that the administered exosomes reduced the progression of CKD in these rats, and this was also evident in the prominent histological improvements in the kidneys of these rats. Other studies targeting TGFβ1 and αSMA in kidney diseases are in accordance with our results [58,59]; however, no studies have used exosomes for the treatment of fibrosis associated with CKD during the menopause period.

MSCs were previously studied in treating diabetic nephropathy, and their favorable effects were attributed to their paracrine factors (either soluble factors or inside extracellular vesicles; as exosomes) [60]. The role of MSCs in repairing kidney injury and fibrosis has been addressed elsewhere [61,62]. However, the use of exosomes for relieving apoptosis and fibrosis in CKD has received less attention in the literature. For example, exosomes isolated from human umbilical cord blood MSCs were found to reduce renal fibrosis induced by mechanical stress through regulating Yes-associated proteins (YAP) in kidney tissue [63] or through ROS-mediated MAPK/ERK signaling [64]. Other studies showed that exosomes isolated from MSCs were able to protect against acute kidney injury [24,65] and reduced autophagy in diabetic nephropathy [66]. In our study, exosomes isolated from bone marrow MSCs were efficient at protecting against post-menopause CKD and their therapeutic use was associated with reduced kidney damage. 

## 5. Conclusions

Our study shows for the first time that exosomes isolated from MSCs have a renal protective role on the kidneys of menopausal rats (used as a model for post-menopause CKD). Exosomes were found to prevent CKD progression by reducing the gene expression levels of NGAL, TGFβ1 and αSMA. In addition, they preserved kidney antioxidant defense mechanisms (GPx, CAT and SOD) and reduced the rate of apoptosis in kidney tissue via reducing the expression of caspase 3 mRNA genes and protein in kidney tissues. This led to stabilizing serum creatinine and BUN levels, reducing urine protein excretion and reducing the damaged histologic picture of the kidneys in exosome-treated rats. Therefore, the current study provides evidence for the utility of exosome therapy in preserving kidney health in post-menopause females and preventing their rapid progression towards CKD.

## Figures and Tables

**Table 1:** Primers used for qRT-PCR.

Gene Name	Sequence (5′-3′)	Accession No.	Ref.
NGAL	F- TTGGGACAGGGAAGACGA R- TCACGCTGGGCAACATTA	XM_032901803.1	[34]
TGFβ1	F- CAGGAGCGCACAATCATGTT R- CTTTAGGAAGGACCTGGGTT	XM_032894155.1	[35]
αSMA	F- GTCCCAGACATCAGGGAGTAA R- TCGGATACTTCAGCGTCAGGA	XM_032891814.1	[35]
Caspase 3	F- CTCGGTCTGGTACAGATGTCGATG R- GGTTAACCCGGGTAAGAATGTGCA	XM_032896303.1	[36]
GAPDH	F- AGACAGCCGCATCTTCTTGT R- TTCCCATTCTCAGCCTTGAC	NM_017008.4	[28]

## Data Availability

The data presented in this study are available in the article.
